# An Online Measurement and Calibration Method for a Radio Telescope Sub-Reflector Support Structure Using Fiber Bragg Grating

**DOI:** 10.3390/mi14051093

**Published:** 2023-05-22

**Authors:** Qian Xu, Hong Bao

**Affiliations:** 1 XinJiang Astronomical Observatory, Chinese Academy of Sciences, Urumqi 830011, China; 2 Key Laboratory of Radio Astronomy, Chinese Academy of Sciences, Urumqi 830011, China; 3 Xinjiang Key Laboratory of Radio Astrophysics, Urumqi 830011, China; 4 Key Laboratory of Electronic Equipment Structure Design, Ministry of Education, Xidian University, Xi’an 710071, China; bh-029@163.com

**Keywords:** sub-reflector, Fiber Bragg Grating (FBG) sensor, self-structuring fuzzy network, measurement model calibration, B-spline functions

## Abstract

The position and altitude of a sub-reflector have an important influence on the pointing accuracy of a radio telescope. With the increase of the antenna aperture, the stiffness of the support structure for the sub-reflector decreases. This causes deformation of the support structure when environmental loads, such as gravity, temperature, and wind load, are applied to the sub-reflector, which will seriously influence antenna pointing accuracy. This paper proposes an online measurement and calibration method for assessing the deformation of the sub-reflector support structure based on the Fiber Bragg Grating (FBG) sensors. Firstly, a reconstruction model between the strain measurements and the deformation displacements of a sub-reflector support structure is established based on the inverse finite element method (iFEM). In addition, a temperature-compensating device with an FBG sensor is designed to eliminate the effects of temperature variations on strain measurements. Considering the lack of the trained original correction, a non-uniform rational B spline (NURBS) curve is built to extend the sample data set. Next, a self-structuring fuzzy network (SSFN) is designed for calibrating the reconstruction model, which can further improve the displacement reconstruction accuracy of the support structure. Finally, a full-day experiment was carried out using a sub-reflector support model to verify the effectiveness of the proposed method.

## 1. Introduction

**Figure 1:**
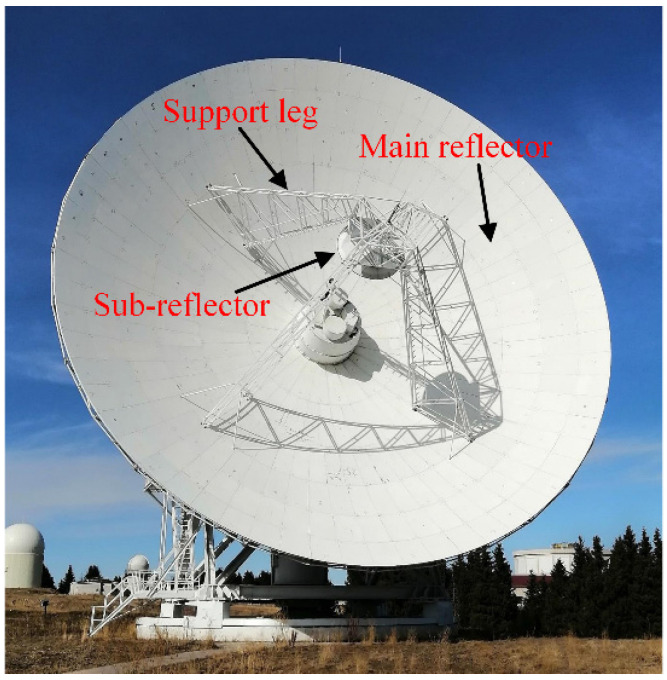
Radio telescope and the sub-reflector.

A sub-reflector is an important part of a large-aperture radio telescope, for it has a direct impact on the electrical performance of the system [1]. Ideally, the position of the sub-reflector relative to the antenna’s main reflector is fixed (Figure 1). However, as the antenna diameter increases, the dimension of the sub-reflector support frame becomes large, and its rigidity decreases. This causes the support frame to deform easily when put under the influence of environmental loads (such as heat, gravity, etc.). A change in the position of the sub-reflector relative to the main reflector will result in a decrease in the electrical performance of the antenna. In particular, this will have a great impact on the pointing accuracy of the antenna. Therefore, the research on the deformation of the sub-reflector support structure under environmental loads has practical significance for maintaining the electrical performance of a radio telescope.

Adjusting the sub-reflector position by an active surface control system is the main method to improve electrical performance [2,3,4]. In this approach, the position and altitude of a deformed sub-reflector support structure must be accurately obtained first in order to provide an accurate input to the active surface control system. Currently, photogrammetry is generally used in engineering for such measurements. However, this method can only be used offline and at night, which means that it only compensates for the deformation caused by gravity. The Sardinian Radio Telescope (SRT) measurement team developed a non-contact measurement scheme based on a position sensing device (PSD) [5], which is composed of a laser diode and a complementary metal oxide semiconductor (CMOS) camera. In this scheme, the accuracy of the PSD can reach about 0.1 mm for a 40 mm measurement range. Owing to the laser capturing capability of the CMOS cameras being easily influenced by the incident angle of the sunlight, this measurement scheme is limited to daytime operation. In order to overcome the above limitations, a contact measurement technique was proposed and applied to large structures [6,7,8]. Compared with traditional strain sensors, Fiber Bragg Grating (FBG) has been widely studied and applied in the field of shape perception because of its lightness, high accuracy, anti-electromagnetic interference, and anti-radiation [9,10]. Marco Bonopera [11] presented important advances in the sensing design and principle of FBG-based displacement sensors. Wang et al. discussed the reliability of the traditional temperature compensation method and proposed a novel temperature compensation method specifically for structures under different loading conditions [12].

The support structure of an antenna sub-reflector is a typical truss structure, which is composed of beam elements. In order to measure the deformation of the beam elements, Ko et al. proposed a load-independent method based on piece-wise continuous polynomials and classical beam theory [13]. The disadvantage of this method is that it only reconstructs structure deformation in one dimension. In practice, it is necessary to measure the deformation of the support structure for the sub-reflector in three dimensions. Bogert et al. analyzed and verified the effectiveness of the modal conversion method by reconstructing structural deformation [14]. Although this method has many advantages, it requires an accurate finite element model. In addition, this method requires a substantial amount of eigenvalue analysis and a detailed description of the properties of the elastic and inertial materials.

To address the problem of inadaptability in the Ko and the modal methods when applying to complex topological structures and boundary conditions during reconstruction, Tessler proposed the inverse finite element method (iFEM) based on the variational principle [15,16]. From the kinematic assumptions of the iFEM frame and Timoshenko beam theory, Gherlone et al. proposed an inverse finite element method for sensing the deformation of a beam or frame structure [17,18]. Bao et al. [19] developed the single variable iFEM model to reduce the number of strain sensors required in multi-node load. Chen et al. [20] established a unified reconstruction method for the beam-like structure based on the framework of iFEM, which can reduce the structure identification error by introducing some generalized quantities. Shang et al. [21] proposed the inverse-plate quadrilateral area coordinates method to replace the Jacobian matrix, such that the error caused by numerical integration can be avoided. Roy et al. [22] expanded the applicability of the iFEM beam model to include prismatic beams with arbitrary cross-sections. Zhao et al. [23] combined the Green-Lagrange strain theory to extend the iFEM for nonlinear deformation.

Due to some uncertain factors, including sensor installation position errors and strain measurement errors presented in actual engineering, the accuracy of shape sensing with the iFEM is affected. Pan et al. proposed to use a fuzzy network to approximate the relationship between the measured strains and the inverse solution strains. They established a fuzzy calibration network through 13 sets of working condition data, and the calibrated strain was used to solve the frame deformation displacement [24]. Fu et al. proposed a fuzzy calibration network with support vectors to generalize the use of the network [25]. The network is obtained by training with 10 sets of working condition data. Since the above-mentioned approaches use less data for training the calibration network, the resulting network covers less information, which will directly influence the accuracy of the calibration network [26]. In addition, Li et al. [27] customize a calibration method using a fuzzy self-framework, which can effectively solve the interference caused by the sensor paste error in iFEM.

However, the reconstruction accuracy of the partial evaluation position was only improved by the above calibration method. In addition, the errors between calibration results and the actual deformation displacement were increased when the faint noise interfered with the working condition data. Considering the reduced influence of external factors on the real-time reconstruction of structural deformation, an online measurement and calibration method for structural deformation based on small samples is proposed in this paper, which can calibrate the reconstruction values of the sub-reflector position and altitude in real-time to improve the reconstruction accuracy. The main original aspects of the proposed method are twofold. Firstly, a reconstruction model between the FBG strain measurements and the deformation displacements of a sub-reflector support structure is established. Secondly, the sample measurement data is extended by the NURBS to provide the training data for the SSFN, which can effectively correct random noise data.

## 2. Establishment of the Deformation Reconstruction Model for Beam Structure

The antenna sub-reflector support legs are a typical truss structure composed of beam elements. This suggests that the inverse finite element beam theory can be used to establish the deformation measurement model for the antenna sub-reflector support structure [17,18]. The measurement procedure is divided into two parts: (1) establishing the reconstruction model with the inverse finite element method and (2) calculating the section strains from the measured strain. This paper proposes a method for temperature compensation to eliminate the effect of temperature on strain measurement.

### 2.1. Inverse Finite Element Method for Beam Element

**Figure 2:**
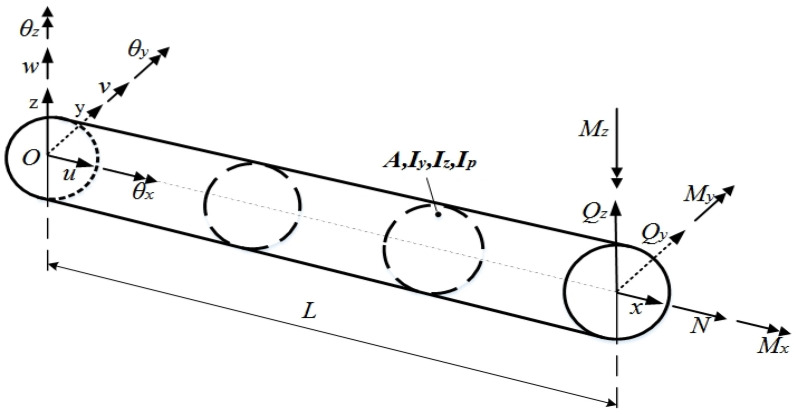
Deformation field of a Timoshenko Round Beam.

Based on the assumption of Timoshenko beam theory, a three-dimensional Cartesian coordinate system for a beam structure of constant cross-section is established, as shown in Figure 2. Here, *L* represents the length of the beam element, the central axis of the beam overlaps with the x-axis of the Cartesian coordinate system, and the y-axis overlaps with the main inertia axis of the section as well. The deformation field at a point B(x,y,z) on the surface can be expressed as

(1) $$\{\begin{array}{c} u_{x}\left(x,\;y,z\right)=u\left(x\right)+z\theta_{y}\left(x\right)-y\theta_{z}\left(x\right)\\ u_{y}\left(x,y,z\right)=v\left(x\right)-z\theta_{x}\left(x\right)\\ u_{z}\left(x,y,z\right)=w\left(x\right)+y\theta_{x}\left(x\right) \end{array},\;$$

where $\left\{u_{x}\left(x,y,z\right),\;u_{y}\left(x,y,z\right),\;u_{z}\left(x,y,z\right)\right\}$ are the deformation displacements along the X, Y, and Z axes at point B, respectively, and $u\left(x,y,z\right)=\{u\left(x\right),v\left(x\right),w\left(x\right),\theta_{x}\left(x\right)$,$\theta_{y}\left(x\right),\theta_{z}\left(x\right)\}$ represents the degree of freedom of the node, as shown in Figure 2.

In the finite element framework, arbitrary nodal degrees of freedom u(x,y,z) can be obtained by the dot product of the shape function N(x) and discrete nodal degrees of freedom $u^{e}$:

(2) $$u\left(x\right)=N\left(x\right)u^{e},$$

Then, the section strain vector $e\left(u^{e}\right)$ can be calculated based on Equation (2), given by

(3) $$e\left(u^{e}\right)=B\left(x\right)u^{e},$$

where B(x) is a matrix containing the derivative of the shape function N(x). However, the theoretical section strain $e\left(u^{e}\right)$ cannot be obtained directly from the strain sensor due to the existence of measurement strain errors. Therefore, a least squares function, φ, is used to establish the relationship between the analytic section strain $e\left(u^{e}\right)$ and measured section strain vector $e^{\varepsilon }$:

(4) $$\varphi \left(u\right)=\|e\left(u^{e}\right)-e^{\varepsilon }\|{}^{2},$$

where $e^{\varepsilon }$ is the calculated section strain from the measured surface strain. Expanding the above least squares error function gives the following quadratic expansion:

(5) $$\varphi \left(u\right)=\frac{1}{2}{\left(u^{e}\right)}^{T}k^{e}u^{e}-{\left(u^{e}\right)}^{T}f^{e}+c^{e},$$

where $c^{e}$ is a constant, and $k^{e}$ and $f^{e}$ are defined as follows:

(6) $$\{\begin{array}{c} k_{k}^{e}=\frac{L}{n}\overset{n}{\underset{i=1}{\sum }}\left[B_{k}^{T}\left(x_{i}\right)B_{k}\left(x_{i}\right)\right]\\ f_{k}^{e}=\frac{L}{n}\overset{n}{\underset{i=1}{\sum }}\left[B_{k}^{T}\left(x_{i}\right)e_{k}^{\varepsilon }\left(x_{i}\right)\right] \end{array},$$

where L is the length of the beam element; n and $x_{i}(0<x_{i}<L)$ are the number and axial coordinates of the estimated section strain position, respectively. When the error function $\varphi^{e}\left(u\right)$ is minimized with respect to ***u^e^***, the result is simplified as

(7) $$k^{e}u^{e}=f^{e}$$

### 2.2. Calculation of Section Strains from Measured Strains

**Figure 3:**
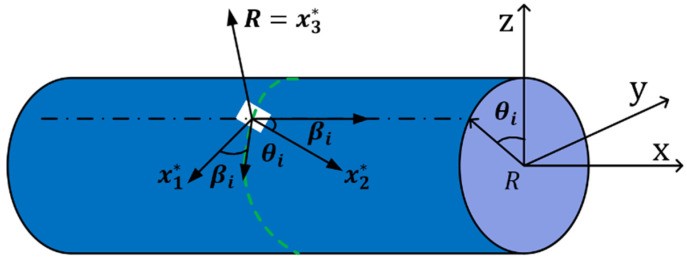
Position coordinates of the beam surface strain sensor.

When the installation position of the strain sensor is determined, the section strain can be calculated from the measured surface strain using the following formula:

(8) $$\begin{array}{c} \varepsilon \left(x_{i},\theta_{i},\beta_{i}\right)=e_{1}^{\varepsilon }\left(x_{i}\right)\left(c_{\beta }^{2}-vs_{\beta }^{2}\right)+e_{2}^{\varepsilon }\left(x_{i}\right)\left(c_{\beta }^{2}-vs_{\beta }^{2}\right)s_{\theta }R+e_{3}^{\varepsilon }\left(x_{i}\right)\left(c_{\beta }^{2}-vs_{\beta }^{2}\right)c_{\theta }R\\ +e_{4}^{\varepsilon }\left(x_{i}\right)c_{\beta }s_{\beta }c_{\theta }-e_{5}^{\varepsilon }\left(x_{i}\right)c_{\beta }s_{\beta }s_{\theta }+e_{6}^{\varepsilon }\left(x_{i}\right)c_{\beta }s_{\beta }R,\\ =\left[\left(c_{\beta }^{2}-vs_{\beta }^{2}\right),\left(c_{\beta }^{2}-vs_{\beta }^{2}\right)s_{\theta }R,\left(c_{\beta }^{2}-vs_{\beta }^{2}\right)c_{\theta }R,c_{\beta }s_{\beta }c_{\theta },c_{\beta }s_{\beta }s_{\theta },c_{\beta }s_{\beta }R\right]\cdot e^{\varepsilon }\left(x_{i}\right)\\ =T\left(x_{i},\theta_{i},\beta_{i}\right)*e^{\varepsilon }\left(x_{i}\right)\\ c_{\beta }\equiv \cos\beta_{i},s_{\beta }\equiv sin\beta_{i},c_{\theta }\equiv cos\theta_{i},s_{\theta }\equiv sin\theta_{i} \end{array}$$

where $x_{i}$ is the coordinates of the *i*-th strain sensor placement, $\theta_{i}$ is the central angle of the section for the *i*-th strain sensor, and $\beta_{i}$ is the angle between the strain measurement direction and the beam element surface generatrix. Therefore, the section strain $e^{\varepsilon }\left(u\right)={\left[e_{1}^{\varepsilon }\left(x_{i}\right),\ldots e_{6}^{\varepsilon }\left(x_{i}\right)\right]}^{T}$ at any section can be determined using Equation (8). In addition, v denotes the Poisson’s ratio, and R stands for the outer diameter of the section, as shown in Figure 3.

### 2.3. Temperature Compensation for Measured Strain

Temperature does not only cause structural deformation but also influences strain measurements. When considering the service environment of a strain sensor, it is also necessary to eliminate the impact of temperature change on the support legs. This paper proposes a real-time temperature compensation method for obtaining the strain after temperature compensation.

Accounting for the influence of the electromagnetic effect from a sensor and the long-distance transmission of measurement information, this paper employs a Fiber Bragg Grating (FBG) sensor as both strain and temperature sensors. Since FBG sensors are based on optical principles and optical fiber material, the advantages of such sensors include the transmission of information without any electromagnetic interference and a low signal attenuation rate.

**Figure 4:**
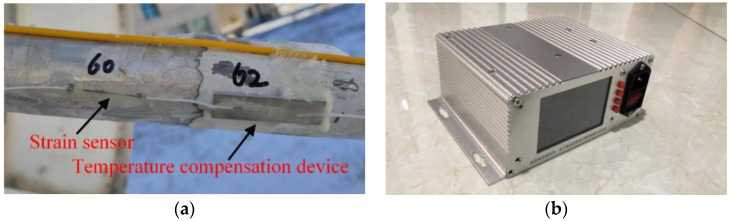
Measurement devices: (**a**) Strain sensor and temperature compensation device; (**b**) Demodulation instrument.

As shown in Figure 4, the Fiber Bragg Grating strain sensor was installed on the surface of the measured object, and a temperature compensation sensor was placed near each strain sensor. It is then followed by connecting the FBG sensors with the demodulation instrument, such that the computer can obtain the strain data from the demodulation instrument. The FBG strain sensor is a novel strain measurement device that is designed to measure the shift in light wavelength caused by the FBG grating deformation due to tension/compressive force or temperature change. The deformation for the unit length of the grating is labeled as the strain, given by

(9) $$\varepsilon_{i}=K\left(\frac{\lambda_{end\left(i\right)}-\lambda_{ini\left(i\right)}}{\lambda_{ini\left(i\right)}}\right)\;,\;K=1-P_{e}$$

where $\lambda_{end\left(i\right)}$ and $\lambda_{ini\left(i\right)}$ are the wavelength shift and initial wavelength of the *i*-th FBG sensor, respectively; P_e_ is the photo-optical coefficient of the fiber; and the strain measurement $\varepsilon_{i}$ is expressed as micro strain.

In Figure 4a, since the FBG strain sensor and temperature compensation sensor are close to each other, they are regarded as possessing the same temperature. The difference between them is in the deformation strain value of the support structure. Meanwhile, the temperature compensation device only measures the strain caused by the environment temperature, which is labeled as $\varepsilon^{comp}$:

(10) $$\varepsilon^{comp}\left(x_{i},\theta_{i},\beta_{i}\right)=\varepsilon \left(x_{i},\theta_{i},\beta_{i}\right)-\varepsilon^{temp}\left(x_{i},\theta_{i},\beta_{i}\right)$$

where the symbol $\varepsilon^{temp}\left(x_{i},\theta_{i},\beta_{i}\right)$ represents the strain value measured by the temperature compensation device.

## 3. The Establishment of Self-Architected Fuzzy Calibration Network

The actual measured displacements at the end of a support leg can be obtained by the measuring device (denoted by $“u_{x}^{act},\;u_{y}^{act},\;u_{z}^{act}”$). Hence, the reconstructed end displacement along different axes is $\mathrm{labeled}\;\mathrm{as}\;“u_{x}^{iFEM},\;u_{y}^{iFEM},\;u_{z}^{iFEM}”$, and their values can be obtained by the inverse finite element method. Hence, the components of the reconstruction errors are

(11) $$\{\begin{array}{c} \Delta u_{x}\left(x_{i},y_{i},z_{i}\right)=u_{x}^{act}\left(x_{i},y_{i},z_{i}\right)-u_{x}^{iFEM}\left(x_{i},y_{i},z_{i}\right)\\ \Delta u_{y}\left(x_{i},y_{i},z_{i}\right)=u_{y}^{act}\left(x_{i},y_{i},z_{i}\right)-u_{y}^{iFEM}\left(x_{i},y_{i},z_{i}\right)\\ \Delta u_{z}\left(x_{i},y_{i},z_{i}\right)=u_{z}^{act}\left(x_{i},y_{i},z_{i}\right)-u_{z}^{iFEM}\left(x_{i},y_{i},z_{i}\right) \end{array}$$

The calibration process is divided into two parts. The first part concerns the construction of a third-order B-spline curve equation based on the measured strain and reconstruction error data. The expansion of the initial data is also performed through Equation (11). The second part involves establishing the self-architected fuzzy calibration network and using the expanded data to realize the real-time calibration of end displacements.

### 3.1. Expansion of Data Sample

The number of samples used to train the self-architected fuzzy network will directly influence the fineness of the network’s description of the relationship between different variables, which in turn determines the precision of the calibration networks. Therefore, expanding the training data sample capacity is of great significance for improving the precision of network calibration. According to the reconstruction theory discussed in Section 2, the relationship between strain measurement and reconstruction displacement is nonlinear. Therefore, this paper proposes to use the 3rd B-spline function for data sample expansion.

For a given *n* + 1 plane or spatial points $P_{i}\left(i=0,1,\cdots ,n\right)$, the definition for an *n*-th parameter segment on a B-spline curve is given by

(12) $$P_{k,n}\left(u\right)=\overset{n}{\underset{i=0}{\sum }}P_{i}G_{i,n}\left(u\right),\;u\epsilon \left[0,1\right]$$

**Figure 5:**
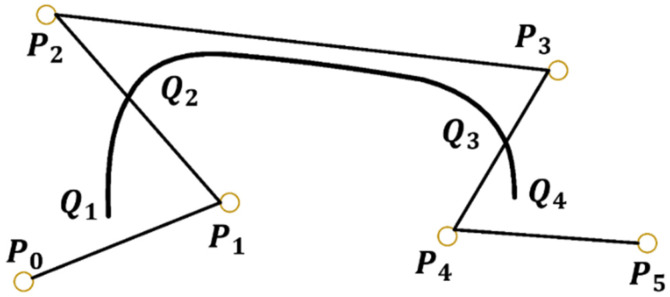
Schematic diagram of cubic B-spline curve.

Here, $P_{k,n}\left(u\right)\;$ is the *k*-th segment on the *n*-th degree B-spline curve, *u* is the data point parameter; $G_{i,n}$ is the basis function of the *n*-th degree B-spline curve, and the polygon formed by its vertex $P_{i}$ is called the B-spline curve feature polygon. According to the definition of the B-spline curve, the k segment of an *n*-th B-spline curve is only related to *n* + 1 control points signified by $P_{i}$. The 3rd B-spline curve is controlled by the 6 control points designated by $P_{i}$ is composed of three B-spline curve segments, each is controlled by 4 points in $P_{i}$, as shown in Figure 5.

The B-spline curve is local. The change of one control point will only influence the adjacent curve segment and will not influence the trend of change on the entire curve. For *n* = 3, the 3rd B-spline curve segment $P_{i,3}\left(u\right)$, is given by

(13) $$P_{i,3}\left(u\right)=\overset{3}{\underset{i=0}{\sum }}P_{i}G_{i,3}\left(u\right),\;u\epsilon \left[0,1\right],$$

where i=0, 1, 2, 3. The basis functions of the cubic B-spline curve are as follows:

(14) $$\{\begin{array}{c} G_{0,3}\left(u\right)=\frac{1}{6}\left(-u^{3}+3u^{2}-3u+1\right)\\ G_{1,3}\left(u\right)=\frac{1}{6}\left(3u^{3}-6u^{2}+4\right)\\ G_{2,3}\left(u\right)=\frac{1}{6}\left(-u^{3}+3u^{2}+3u+1\right)\\ G_{3,3}\left(u\right)=\frac{1}{6}u^{3} \end{array},\;u\epsilon \left[0,1\right]$$

It can be written in matrix form as

(15) $$\{\begin{array}{c} x_{u}=GX\\ y_{u}=GY \end{array},\;\;\;\;\;\;u\epsilon \left[0,1\right]$$

where G is the basis function matrix of the cubic B-spline, *X* and *Y* are the coordinate vectors of the control points in each section of the cubic B-spline curve, and $\left(x_{u},y_{u}\right)$ are the coordinates of a point on the cubic B-spline curve. For continuous values of u, a smooth B-spline curve can be determined. For *n* control vertices, a complete cubic B-spline curve can be obtained by only moving the control vertices *n*−3 times. After successfully fitting the cubic B-spline curve, the data point parameter *u* is taken from 0 to 1 in a certain step, such that the corresponding number of points on the curve can be obtained, and the data can be expanded.

### 3.2. Construction of the Calibration Network

In reality, there are errors in the sensor installation and data collection, which lead to subsequent displacement reconstruction errors. In this paper, the self-architected fuzzy network (SAFN) algorithm is used to calibrate the reconstruction errors. The algorithm uses the triangular membership functions (MF), which independently increases the membership functions and fuzzy rules together with adjusting their distributions, thereby improving the structure of the fuzzy system. Based on the cubic B-splines, large-scale data is obtained, and a further fuzzy network is derived to approximate the measured strain and reconstruction error.

**Figure 6:**
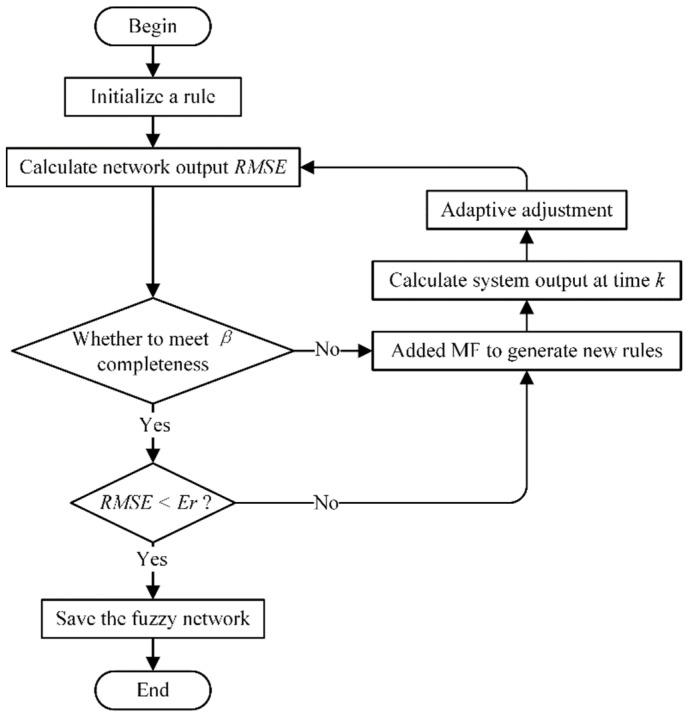
Flowchart of the self-structuring fuzzy network algorithm.

The generation of self-architected fuzzy networks can be divided into three parts: (1) adding MF and generating the rules, (2) establishing self-adapting fuzzy rules and consequent parts, and (3) preserving fuzzy networks [24]. The algorithm flowchart is shown in Figure 6.

1. Adding MF and generation rules
  (1) Error criterion

The root-mean-square error is used to describe the system error, and its calculation is given by Equation (16). In the equation, y(k) represents the output value from the fuzzy network, and $y_{d}\left(k\right)$ signifies the reconstruction deformation error. Hence, the system error $RMSE_{2}$ can be calculated. The symbol $E_{r}$ represents the error threshold in the training phase. If $RMSE_{2}>E_{r}$, the membership function needs to be increased at the input.

(16) $$RMSE=\sqrt{\frac{\sum_{k=1}^{N}{\left(y\left(k\right)-y_{d}\left(k\right)\right)}^{2}}{N}}$$

  
  (2) Completeness criteria

For any input variable $x_{j}$ in the working range, the current value $x_{j}\left(k\right)$ can help activate an MF, and the maximum value for $\mu_{m}\left(x_{j}\left(k\right)\right)$ of the obtained membership degree is either greater than or equal to the preset β value. If $\mu_{m}\left(x_{j}\left(k\right)\right)<\beta$, MF needs to be increased or is otherwise unchanged.

2. The consequence of self-adaptive rules

Based on the current estimated output value and the expected output value from the fuzzy network, the rule consequences are adjusted. At *k* moment, the expression for adjusting the consequence of the *j*-th rule $\alpha_{j}\left(k\right)$ is as follows:

(17) $$\Delta \alpha_{j}\left(k\right)=\gamma \cdot \mu_{j}\left(k-1\right)\cdot \left(r\left(k-1\right)-y\left(k\right)\right),$$

where $\mu_{j}\left(k-1\right)$ represents the activation of the *j*-th rule at time k−1, r(k−1) represents the estimated displacement that is input to the network at the previous time, and y(k) is the calibrated displacement error output from the fuzzy network at the current time. The value of γ is artificially adjusted to change the speed of the self-adaptive process of the rule consequence.

3. Fixing the rules and obtaining the fuzzy network

After adding the data, the estimated output error from the network for all training input values becomes very small. For $E_{FN}<E_{r}$, it means that the autonomous architecture stage of the network is completed. The network training is then stopped, and the fuzzy rules in the current network are saved.

After expanding the samples of strain and reconstruction error through the cubic B-spline theory, the expanded samples are used to train the fuzzy network and generate the fuzzy calibration network. In engineering practice, when the real-time measured strain $\varepsilon \left(x_{i},\theta_{i},\beta_{i}\right)\;$ is input into the fuzzy calibration network, the calibrated displacement error value can be presented in real-time. After that, the accurately reconstructed displacements will be obtained.

## 4. Experimental Examples

**Figure 7:**
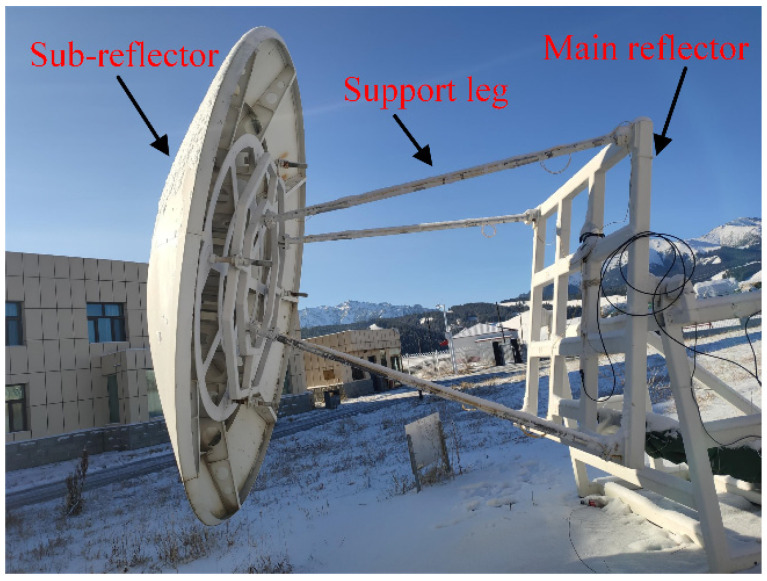
Scaled-down model for the radio telescope test antenna.

In order to verify the accuracy and effectiveness of the online reconstructed displacement error calibration method presented in this paper, a scaled-down model for a radio telescope test antenna model was constructed, as shown in Figure 7. Under the elevation motion, the support structure’s deformation measurements and calibration experiment were carried out. The support structure is made of aluminum alloy material. Its Young’s modulus is 7300 MPa, Poisson’s ratio is v=0.3, density $\rho =2557\;\mathrm{kg}/m^{3}$, and the entire structure weighs about 43 kg. The support beam is 2000 mm long and has a radius of 20 mm. The total weight of the sub-reflector is 200 kg.

In order to realize the deformation reconstruction for the support legs of the sub-reflector, six fiber grating strain sensors are installed on the surface of each support leg, and three sensors are placed on the same section to capture the surface strain. In the experiments, the strain data is measured by the FBG strain sensor (Fiber Bragg Grating| os1100, Micron Optics, Atlanta, GA, USA) and the FBG demodulator (Optical Sensing Instrument| Si 155, Micron Optics, Atlanta, GA, USA) Strain measurement system. The positions of the six strain sensors are shown in Table 1. Here, $x_{k}$ represents the relative position, and (θ,β) are the circumferential angle θ with angle β between x-axis and the frame, both measured in degrees. The properties of the FBG sensor include 1530–1565 wavelength operating range, 0.23 nm bandwidth, 15 db side-mode rejection ratio, 10 mm grid length, and 1 pm demodulation accuracy.

**Figure 8:**
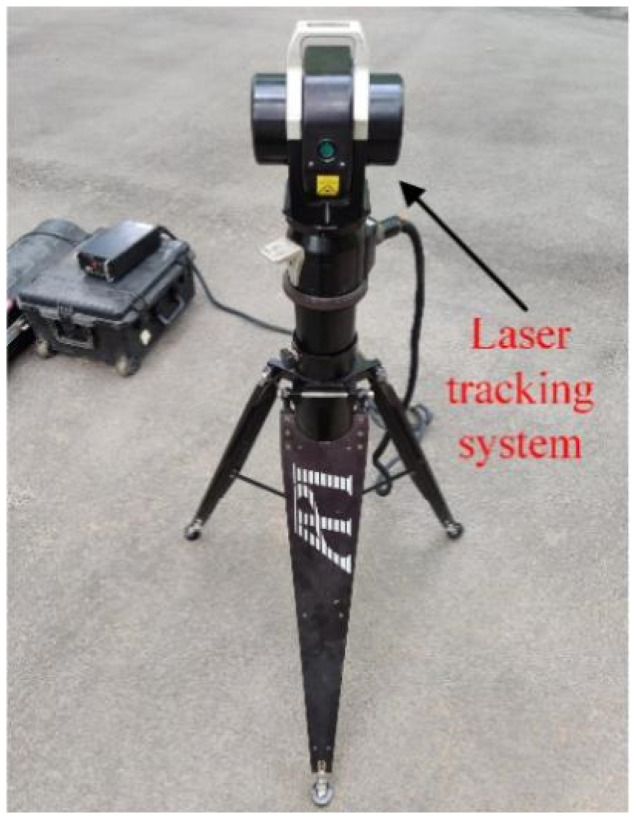
Deformation measurement system.

A high-precision single-point laser tracker (LTS, API Tracker 3, Automated Precision Inc., Rockwell, MD, USA) was used to measure the actual displacement at the end of the support leg and at different elevation angles. The resolution of the LTS is 1 um, and the precision is related to the distance between the LTS and the measured object (the ratio is 5 um/m). During the experiment, the distance between the instrument and the test antenna model was 6 m, and the measurement precision of the laser tracker was 0.03 mm. The entire measurement system is shown in Figure 8.

**Figure 9:**
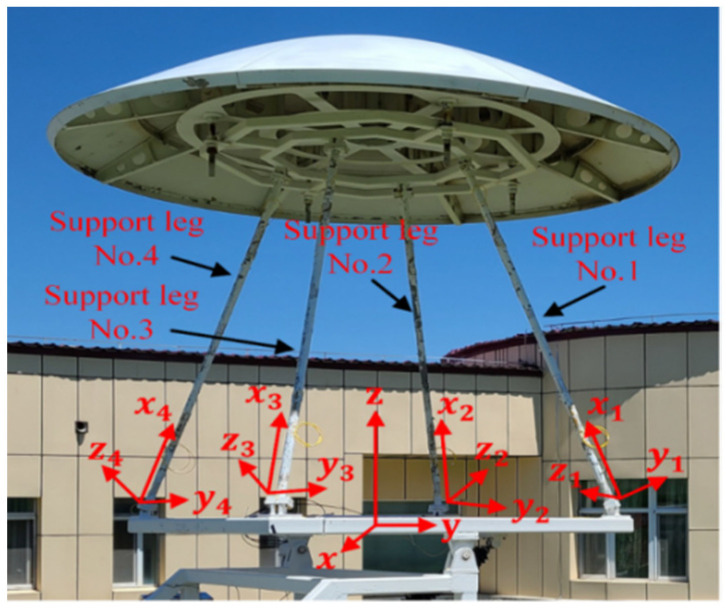
Global and local coordinate systems.

In the experiments, the global coordinate system was established on the main reflector. In each local coordinate system, the x-axis was established along the neutral axis of each support beam, the y-axis and z-axis were orthogonal in the beam section, and the four support beams had four different local coordinate systems (Figure 9).

**Figure 10:**
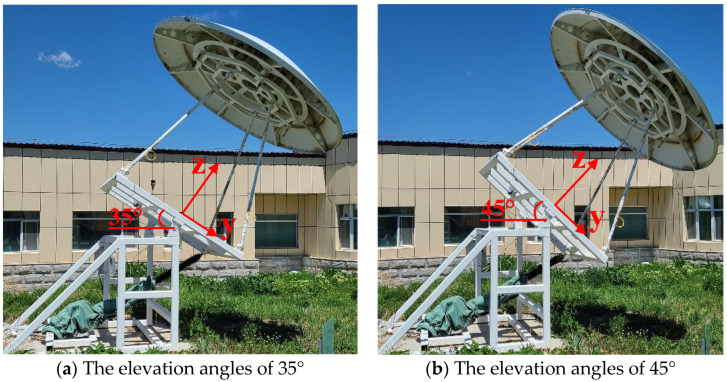
Test antenna model at two different elevation angles.

In order to better validate the effectiveness of the calibration scheme proposed in this paper, the strain and real displacement data at the end of the support leg at each elevation angle were collected at midnight to ensure that the model was in a stable environmental temperature. Assuming the initial position and altitude of 0 degree between the main reflector of the antenna and the horizontal plane, the strain data collected by the fiber demodulator was recorded and returned to zero. The platform was then rotated and stopped every 5 degrees increment to measure the strains and true displacements of the support leg (see Figure 10).

From 5 to 60 degrees, the strains $\varepsilon^{*}$ and displacements at the end of the support legs $\left\{u_{x}^{act},\;u_{y}^{act},\;u_{z}^{act}\right\}$ were collected. As shown in the second section, the reconstruction value $\left\{u_{x}^{iFEM},\;u_{y}^{iFEM},\;u_{z}^{iFEM}\right\}$ of the deformation at the end of the support legs can be calculated by $\varepsilon \left(x_{i},\theta_{i},\beta_{i}\right)$. According to Equation (3), the reconstruction errors can be obtained in the form of $\left\{\Delta u_{x},\;\Delta u_{y},\;\Delta u_{z}\right\}$. From the 12 working conditions, the data at the elevation angles of $15^{{}^{\circ}}$, $30^{{}^{\circ}}$, $40^{{}^{\circ}}$, and $55^{{}^{\circ}}$ were used to test the precision of the calibration network, and the data of the remaining eight working conditions were used to train the self-architected fuzzy calibration network.

For each support leg, the strain $\varepsilon^{*}$ that shows the greatest reaction to changes of the elevation angle in the model is selected and combined with the reconstruction error to form three control points of deformation direction, designated by $\left\{\varepsilon^{*},\;\Delta u_{x}\left(x_{k},y_{k},z_{k}\right)\right\}$, $\left\{\varepsilon^{*},\;\Delta u_{y}\left(x_{k},y_{k},z_{k}\right)\right\}$ and $\left\{\varepsilon^{*},\Delta u_{z}\left(x_{k},y_{k},z_{k}\right)\right\},\;\mathrm{with}\;\left(k=1,2\cdots 8\right)$. Consider $\left\{\varepsilon^{*},\;\Delta u_{x}\left(x_{k},y_{k},z_{k}\right)\right\}$, the eight working conditions form eight vertices. According to Section 3.1, the cubic B-spline curve can be obtained. The data from the eight groups of working conditions can be expanded based on the value in the parameter u from 0 to 1, in step of 0.002, thereby forming 501 sets of data. Similarly, for $\left\{\varepsilon^{*},\;\Delta u_{y}\left(x_{k},y_{k},z_{k}\right)\right\}$ and $\left\{\varepsilon^{*},\Delta u_{z}\left(x_{k},y_{k},z_{k}\right)\right\}$, which are also expanded into 501 sets of data. Using the extended data from a single direction of the support leg for training, three sets of fuzzy calibration networks, $SSFN_{x}$, $SSFN_{y}$, and $SSFN_{z}$, can be obtained. This is then followed by inputting the measured strains, obtained at the elevation angles of $15^{{}^{\circ}}$, $30^{{}^{\circ}}$,$40^{{}^{\circ}}$, and $55^{{}^{\circ}}$, into each $SSFN_{x}$, $SSFN_{y}$, and $SSFN_{z}$ to determine the calibrated displacement error. Finally, the calibration displacement value is obtained by adding the calibration error to the initial reconstruction displacement.

When the fuzzy calibration network is completed, the real-time input strain and output deformation calibration can be realized. In this paper, four elevation angles taken at midnight were used as a case study to validate the calibration network. For the support 1, the displacement and calibration error as shown in Table 2, and corresponding FBG strains and temperature compensation as shown in Table 3. Meanwhile, the estimated index of the fuzzy calibration network’s ability defined as

(18) $$\delta \left(\%\right)=\frac{\left|u^{act}-u^{cal}\right|}{u^{act}}\times 100\%$$

the actual, reconstructed, and calibrated displacements are denoted as $u^{act}$, $u^{iFEM}$ and $u^{cal}$.

For the support 2, the displacement and calibration error as shown in Table 4, and corresponding FBG strains and temperature compensation as shown in Table 5.

For the support 3, the displacement and calibration error as shown in Table 6, and corresponding FBG strains and temperature compensation as shown in Table 7.

For the support 4, the displacement and calibration error as shown in Table 8, and corresponding FBG strains and temperature compensation as shown in Table 9.

It can be seen from Table 2, Table 3, Table 4, Table 5, Table 6, Table 7, Table 8 and Table 9 that the reconstructed displacements for the end of the support legs can be calibrated well at different elevation angles using the small sample self-architected fuzzy calibration algorithm proposed in this paper. The percentage displacement errors in the x, y, and z directions do not exceed 4% after calibrating the four support legs. In particular, for the elevation angle at 40 degrees, the y-direction displacement percentage error at the end of the No. 1 support leg is 0%. It can be seen from the actual displacements in the x, y, and z directions that the y direction is the main deformation direction. In most cases, the calibration error in the y direction is less than that in the *x* and *z* directions when the deformation is large. This suggests that the presented calibration scheme in this paper has better calibration ability for the main deformation direction.

In order to further verify the effectiveness of the temperature compensation device designed in this paper, the strain data under the elevation of 55° at noon and from the No. 1 support leg was used. In this case, the strain value $\varepsilon^{comp}\left(x_{i},\theta_{i},\beta_{i}\right)$ was obtained by Equation (10). It can be seen from Table 10 that the temperature has a great influence on strain measurement. However, when the measured strains, ε, are compensated, $\varepsilon^{comp}$ becomes stable.

ε$\varepsilon^{temp}$$\varepsilon^{comp}$ Comparing the displacement reconstruction value $u^{PM}$, $u^{iFEM}$ at noon and at midnight with the displacement $u^{comp}$, the results are as follows.

It can be seen from Table 11 that the displacement reconstruction value at the end of No. 1 support leg measured at noon has a deviation of 0.67 mm in the *y* direction when compared with the measurement taken at night. The deviation decreases to 0.06 mm after temperature compensation for the measured strain. It is clear that the temperature compensation method can greatly reduce the influence of environmental temperature changes on the strain acquisition system. Therefore, the self-structuring fuzzy network and the temperature compensation device proposed in this paper are important for improving the accuracy of traditional inverse finite elements in real-world engineering applications.

## 5. Conclusions

We have proposed an online measurement and calibration method for deformation estimation for the support structure of a sub-reflector in order to provide accurate adjustment data for the sub-reflector control system of a radio telescope. Firstly, the fiber grating sensors are used to measure the surface strain of the support leg. It is then followed by deformation reconstruction for the end of the support leg of the sub-reflector using the inverse finite element method. Finally, the proposed calibration method is used to calibrate the reconstruction displacement to improve the precision. The results show that the percentage error for each support leg is less than 4%, with the minimum error reaching 0%. Comparing the calibration results along the three directions, we demonstrate that our calibration method is accurate along the main deformation direction. For problems relating to small sample data, this paper proposes the use of the cubic B-spline to expand the initial working condition data so as to provide a data guarantee for the training of a high-precision calibration network. In addition, a temperature compensation device is designed to compensate the measured strains in order to reduce the influence of ambient temperature on reconstruction accuracy. The experimental results show that the device can effectively reduce the influence of service temperature on the reconstruction. Furthermore, the demonstrated calibration method can be applied to other structures, such as large reflectors, for deformation measurement. However, our experimental period was short, and further exploration is required for the long-term application of the temperature compensation device over wide temperature ranges.

## Figures and Tables

**Table 1:** Strain sensor locations.

Axial Position $x_{k}$	0.2*L*	0.8*L*
$\left(\theta_{1},\beta_{1}\right)$	(0,0)	(0,45)
$\left(\theta_{2},\beta_{2}\right)$	(120,0)	(−120,0)
$\left(\theta_{3},\beta_{3}\right)$	(−120,0)	(120,0)

**Table 2:** Displacement and calibration error for No. 1 support leg.

Angles		x Direction	y Direction	z Direction
$55^{{}^{\circ}}$	$u^{act}$	1.86 mm	19.01 mm	4.76 mm
	$u^{iFEM}$	1.53 mm	17.48 mm	4.56 mm
	$u^{cal}$	1.83 mm	18.79 mm	4.71 mm
	δ(%)	1.61%	1.16%	1.05%
$40^{{}^{\circ}}$	$u^{act}$	1.56 mm	15.85 mm	3.99 mm
	$u^{iFEM}$	1.26 mm	14.18 mm	3.71 mm
	$u^{cal}$	1.54 mm	15.85 mm	3.98 mm
	δ(%)	1.28%	0%	0.25%
$30^{{}^{\circ}}$	$u^{act}$	1.29 mm	12.70 mm	3.25 mm
	$u^{iFEM}$	0.97 mm	10.75 mm	2.85 mm
	$u^{cal}$	1.28 mm	12.68 mm	3.22 mm
	δ(%)	0.78%	0.16%	0.92%
$15^{{}^{\circ}}$	$u^{act}$	0.75 mm	6.66 mm	1.77 mm
	$u^{iFEM}$	0.48 mm	5.15 mm	1.41 mm
	$u^{cal}$	0.74 mm	6.49 mm	1.73 mm
	δ(%)	1.33%	2.55%	2.26%

**Table 3:** FBG strains and temperature compensation for No. 1 support leg.

Angles		$55^{{}^{\circ}}$	$40^{{}^{\circ}}$	$30^{{}^{\circ}}$	$15^{{}^{\circ}}$
**FBG strain/με**	$\varepsilon_{1}$	−20	−62	−101	−173
	$\varepsilon_{2}$	−485	−431	−376	−263
	$\varepsilon_{3}$	−111	−115	−117	−112
	$\varepsilon_{4}$	−345	−314	−272	−191
	$\varepsilon_{5}$	418	331	251	96
	$\varepsilon_{6}$	−79	−82	−86	−91
**FBG temperature compensation/με**	$\varepsilon_{1}$	55	52	51	43
	$\varepsilon_{2}$	21	25	32	33
	$\varepsilon_{3}$	28	34	39	37
	$\varepsilon_{4}$	7	13	20	25
	$\varepsilon_{5}$	29	36	37	34
	$\varepsilon_{6}$	27	31	36	33

**Table 4:** Displacement and calibration error for No. 2 support leg.

Angles		x Direction	y Direction	z Direction
$55^{{}^{\circ}}$	$u^{act}$	1.67 mm	16.76 mm	4.66 mm
	$u^{iFEM}$	1.40 mm	18.86 mm	6.35 mm
	$u^{cal}$	1.63 mm	16.53 mm	4.68 mm
	δ(%)	2.4%	1.37%	0.43%
$40^{{}^{\circ}}$	$u^{act}$	1.40 mm	14.00 mm	3.90 mm
	$u^{iFEM}$	1.16 mm	15.53 mm	5.22 mm
	$u^{cal}$	1.38 mm	13.97 mm	3.94 mm
	δ(%)	1.43%	0.21%	1.03%
$30^{{}^{\circ}}$	$u^{act}$	1.16 mm	11.38 mm	3.14 mm
	$u^{iFEM}$	0.89 mm	12.04 mm	4.08 mm
	$u^{cal}$	1.15 mm	11.36 mm	3.11 mm
	δ(%)	0.86%	0.18%	0.96%
$15^{{}^{\circ}}$	$u^{act}$	0.68 mm	6.12 mm	1.57 mm
	$u^{iFEM}$	0.44 mm	6.02 mm	2.08 mm
	$u^{cal}$	0.67 mm	6.01 mm	1.53 mm
	δ(%)	1.47%	1.8%	2.55%

**Table 5:** FBG strains and temperature compensation for No. 2 support leg.

Angles		$55^{{}^{\circ}}$	$40^{{}^{\circ}}$	$30^{{}^{\circ}}$	$15^{{}^{\circ}}$
**FBG strain/με**	$\varepsilon_{1}$	−237	−237	−238	−227
	$\varepsilon_{2}$	−484	−438	−386	−282
	$\varepsilon_{3}$	−17	−43	−66	−112
	$\varepsilon_{4}$	−128	−122	−114	−105
	$\varepsilon_{5}$	365	283	206	62
	$\varepsilon_{6}$	−560	−485	−404	−252
**FBG temperature** **compensation/με**	$\varepsilon_{1}$	22	26	27	30
	$\varepsilon_{2}$	16	19	26	30
	$\varepsilon_{3}$	25	27	31	32
	$\varepsilon_{4}$	−8	1	12	18
	$\varepsilon_{5}$	29	34	37	35
	$\varepsilon_{6}$	9	14	19	23

**Table 6:** Displacement and calibration error for No. 3 support leg.

Angles		x Direction	y Direction	z Direction
$55^{{}^{\circ}}$	$u^{act}$	2.29 mm	17.10 mm	−0.73 mm
	$u^{iFEM}$	−0.18 mm	15.77 mm	−0.84 mm
	$u^{cal}$	2.22 mm	16.89 mm	−0.72 mm
	δ(%)	3.06%	1.23%	1.37%
$40^{{}^{\circ}}$	$u^{act}$	1.90 mm	14.25 mm	−0.61 mm
	$u^{iFEM}$	−0.25 mm	12.79 mm	−0.69 mm
	$u^{cal}$	1.88 mm	14.26 mm	−0.60 mm
	δ(%)	1.05%	0.07%	1.64%
$30^{{}^{\circ}}$	$u^{act}$	1.48 mm	11.42 mm	−0.48 mm
	$u^{iFEM}$	−0.34 mm	9.70 mm	−0.53 mm
	$u^{cal}$	1.51 mm	11.39 mm	−0.47 mm
	δ(%)	2.03%	0.26%	2.08%
$15^{{}^{\circ}}$	$u^{act}$	0.78 mm	5.99 mm	−0.25 mm
	$u^{iFEM}$	−0.29 mm	4.65 mm	−0.26 mm
	$u^{cal}$	0.81 mm	5.92 mm	−0.24 mm
	δ(%)	3.85%	1.17%	4.0%

**Table 7:** FBG strains and temperature compensation for No. 3 support leg.

Angles		$55^{{}^{\circ}}$	$40^{{}^{\circ}}$	$30^{{}^{\circ}}$	$15^{{}^{\circ}}$
**FBG strain/με**	$\varepsilon_{1}$	−16	−32	−50	−74
	$\varepsilon_{2}$	−369	−335	−295	−212
	$\varepsilon_{3}$	30	4	−21	−61
	$\varepsilon_{4}$	−187	−180	−165	−138
	$\varepsilon_{5}$	328	240	164	25
	$\varepsilon_{6}$	−359	−309	−261	−173
**FBG temperature compensation/με**	$\varepsilon_{1}$	14	22	28	28
	$\varepsilon_{2}$	17	20	26	29
	$\varepsilon_{3}$	27	30	38	34
	$\varepsilon_{4}$	9	18	24	27
	$\varepsilon_{5}$	35	36	38	36
	$\varepsilon_{6}$	12	21	25	29

**Table 8:** Displacement and calibration error for No. 4 support leg.

Angles		x Direction	y Direction	z Direction
$55^{{}^{\circ}}$	$u^{act}$	1.93 mm	16.90 mm	−1.52 mm
	$u^{iFEM}$	−0.22 mm	18.91 mm	−1.39 mm
	$u^{cal}$	1.88 mm	16.70 mm	−1.50 mm
	δ(%)	2.59%	1.18%	1.32%
$40^{{}^{\circ}}$	$u^{act}$	1.60 mm	14.12 mm	−1.27 mm
	$u^{iFEM}$	−0.27 mm	15.57 mm	−1.13 mm
	$u^{cal}$	1.58 mm	14.11 mm	−1.26 mm
	δ(%)	1.25%	0.07%	0.79%
$30^{{}^{\circ}}$	$u^{act}$	1.25 mm	11.47 mm	−1.03 mm
	$u^{iFEM}$	−0.33 mm	12.07 mm	−0.86 mm
	$u^{cal}$	1.26 mm	11.39 mm	−1.02 mm
	δ(%)	0.80%	0.70%	0.97%
$15^{{}^{\circ}}$	$u^{act}$	0.66 mm	6.17 mm	−0.55 mm
	$u^{iFEM}$	−0.27 mm	6.02 mm	−0.41 mm
	$u^{cal}$	0.67 mm	6.07 mm	−0.54 mm
	δ(%)	1.52%	1.62%	1.82%

**Table 9:** FBG strains and temperature compensation for No. 4 support leg.

Angles		$55^{{}^{\circ}}$	$40^{{}^{\circ}}$	$30^{{}^{\circ}}$	$15^{{}^{\circ}}$
**FBG strain**	$\varepsilon_{1}$	13	−9	−30	−73
	$\varepsilon_{2}$	−369	−338	−299	−220
	$\varepsilon_{3}$	26	−1	−21	−61
	$\varepsilon_{4}$	−191	−176	−165	−140
	$\varepsilon_{5}$	−288	−272	−239	−184
	$\varepsilon_{6}$	421	337	258	100
**FBG temperature** **compensation**	$\varepsilon_{1}$	11	10	18	28
	$\varepsilon_{2}$	21	21	27	30
	$\varepsilon_{3}$	35	36	37	36
	$\varepsilon_{4}$	5	8	15	23
	$\varepsilon_{5}$	37	39	42	34
	$\varepsilon_{6}$	18	24	27	27

**Table 10:** Two strain measurements for No. 1 support leg at $55^{{}^{\circ}}$.

Strain	Time	$\varepsilon_{1}/\mu \varepsilon$	$\varepsilon_{2}/\mu \varepsilon$	$\varepsilon_{3}/\mu \varepsilon$	$\varepsilon_{4}/\mu \varepsilon$	$\varepsilon_{5}/\mu \varepsilon$	$\varepsilon_{6}/\mu \varepsilon$
ε	Midnight	−20.48	−484.92	−111.42	−345.13	417.76	−79.67
	Noon	−327.20	−601.49	−118.92	−354.49	45.15	−125.02
$\varepsilon^{temp}$	Midnight	353.65	−315.15	48.26	−152.23	616.02	86.15
	Noon	52.94	−442.72	38.77	−150.58	246.41	45.80
$\varepsilon^{comp}$	Midnight	−374.14	−169.77	−159.69	−192.91	−198.26	−165.83
	Noon	−380.14	−158.77	−157.69	−203.91	−201.26	−170.83

**Table 11:** Displacement o No. 1 support leg at $55^{{}^{\circ}}$.

Reconstruction Value	x Direction/mm	y Direction/mm	z Direction/mm
$u^{iFEM}$	1.53	17.48	4.56
$u^{PM}$	1.47	18.15	4.73
$u^{comp}$	1.51	17.42	4.54

## Data Availability

Not applicable.
